# Distribution and concordance of PD-L1 expression by routine 22C3 assays in East-Asian patients with non-small cell lung cancer

**DOI:** 10.1186/s12931-022-02201-8

**Published:** 2022-11-05

**Authors:** Fangqiu Fu, Chaoqiang Deng, Wenrui Sun, Qiang Zheng, Yan Jin, Yuan Li, Yang Zhang, Haiquan Chen

**Affiliations:** 1 Department of Thoracic Surgery and State Key Laboratory of Genetic Engineering, Fudan University Shanghai Cancer Center, 270 Dong-An Road, Shanghai, 200032 China; 2 Institute of Thoracic Oncology, Fudan University, Shanghai, 200032 China; 3 Department of Oncology, Shanghai Medical College, Fudan University, Shanghai, 200032 China; 4 Department of Pathology, Fudan University Shanghai Cancer Center, Shanghai, 200032 China

**Keywords:** PD-L1, Non-small cell lung cancer, Distribution, Concordance

## Abstract

**Background:**

Currently, programmed death ligand-1 (PD-L1) expression has been widely applied in clinical trials and real-world clinical practice as a major biomarker for the efficacy of immune-checkpoint inhibitors. The purpose of this study is to reveal the distribution and concordance of PD-L1 expression in a large-scale consecutive cohort from East-Asian patients with non-small cell lung cancer (NSCLC).

**Methods:**

PD-L1 testing was conducted using 22C3 assays, and cases were categorized into the high, low, and no expression of PD-L1 based on the tumor proportion score (TPS). Target-capture next-generation sequencing was used to identify molecular events.

**Results:**

A total of 4550 patients and 4622 tests of PD-L1 expression were enrolled. There were 3017 (66.3%) patients with no PD-L1 expression (TPS < 1%), 1013 (22.3%) with low PD-L1 expression (TPS 1–49%), 520 (11.4%) with high PD-L1 expression (TPS ≥ 50%). Higher proportions of positive PD-L1 expression (TPS ≥ 1%) were observed in smokers, males, squamous cell carcinoma, and high-grade lung adenocarcinoma. Further analyses revealed fair agreement in primary and metastatic lesions (kappa = 0.533), poor agreement in multi-focal primary tumors (kappa = 0.045), and good agreement in biopsy and resection samples (kappa = 0.662) / two biopsy samples (kappa = 0.711). Mutational analyses revealed association between high PD-L1 expression (TPS ≥ 50%) and *EGFR* wild-type, *KRAS* mutation, *ALK* rearrangement, and *TP53* mutation.

**Conclusion:**

The study reveals the unique distribution pattern of PD-L1 expression in a large-scale East-Asian cohort with NSCLC, the concordance of multiple PD-L1 tests, and the association between PD-L1 expression and molecular events. The results shed a light on the optimization of PD-L1 testing in clinical practice.

**Supplementary Information:**

The online version contains supplementary material available at 10.1186/s12931-022-02201-8.

## Introduction

Immune-checkpoint inhibitors (ICIs) towards programmed cell death protein-1 (PD-1)/ programmed death ligand-1 (PD-L1) have revolutionized the treatments of lung cancer and substantially elevated the survival of patients with lung cancer. Recently, the five-year outcomes of KEYNOTE-024 were reported [1], and pembrolizumab (PD-1 inhibitor) could significant improved overall survival (OS) by 38% and progression-free survival (PFS) by 50% in patients with metastatic non-small lung cancer (NSCLC) with PD-L1 tumor proportion score (TPS) ≥ 50% compared with chemotherapy. Meanwhile, despite long-term OS benefit provided by PD-1/PD-L1 ICIs, only 15–25% of patients with NSCLC will respond initially in real-world clinical practice [2]. Therefore, there is an urgent need to find an effective way to identify subgroups of patients who will benefit from ICIs.

There are several soluble predictive biomarkers of response to ICIs, such as PD-L1 expression, tumor mutation burden, specific tumor mutation (e.g. mutations in DNA replication or repair genes), and tumor-associated immune cell (e.g. CD8+ T cells) [3–5]. Although imperfect, PD-L1 expression stands out above the rest for its good performance and clinical feasibility. Although immunohistochemistry (IHC) of PD-L1 expression has been widely applied clinically [2], there remains a lack of published studies on the prevalence of PD-L1 expression in a large-scale eastern-Asian cohort and the concordance of PD-L1 expression in patients with multiple lung cancers or repeated PD-L1 testing. In addition, previous studies mainly focus on advanced lung cancer using biopsy specimens, which could not fully represent the whole picture of tumor, leading to deviations of results.

In this study, we reported real-world prevalence and concordance of PD-L1 expression using 22C3 assays in a consecutive population-based East-Asian NSCLC cohort mainly using surgical specimens. Our study revealed the concordance of PD-L1 testing from multiple biopsies/samples, as well as the association between PD-L1 expression and common clinicopathological factors, including gene mutation status, which provided insights into PD-L1 testing for patients with NSCLC.

## Materials and methods

### Patients

This study was approved by the Institutional Review Board of Fudan University Shanghai Cancer Center (FUSCC) (IRB#090977-1) under the approval number of 2008223-9, and it was carried out in a consecutive NSCLC cohort who received surgical resection or biopsy at the Department of Thoracic Surgery and PD-L1 testing at the Department of Pathology in Fudan Shanghai Cancer Center from September 2017 to April 2021. The following clinicopathological factors were prospectively collected: age, sex, smoking history, sample type, tumor histology, TNM stage, tumor spread thorough air space (STAS), and common gene alterations. The distinguishment of multifocal or metastatic lung cancers was based on the criteria released by IASLC Lung Cancer Staging Project [6].

### Testing and evaluation of PD-L1 expression

PD-L1 testing was performed on formalin-fixed paraffin-embedded (FFPE) samples within 1 week after surgery. IHC of PD-L1 was conducted by 22C3 assays (Agilent Technologies) using the Dako Autostainer Link 48 platform following its manufacturer’s instructions. Tumor proportion score (TPS) of PD-L1 expression were defined as the percentage of tumor cells with positive PD-L1 staining over all tumor cells. Specimens containing less than 100 measurable tumor cells were excluded. PD-L1 expression was further categorized into no expression (TPS < 1%), low expression (TPS: 1–49%), and high expression (TPS ≥ 50%). Specimen slides were reviewed by two experienced pulmonary pathologists (Y. J. and Y. L.). Any disagreements were resolved by re-review and discussion until agreements were reached.

### Gene alteration testing using next-generation sequencing

Target-capture next-generation sequencing (NGS) was conducted with genomic DNA using a 68-gene panel (Burning Rock Inc., China). DNA was extracted using QIAamp DNA FFPE tissue kit (QIAGEN, Germany) following the manufacturer’s instructions. DNA was sheared by the Covaris M220 instrument (Covaris Inc., Woburn, MA, USA), followed by end repair and adaptor ligation. Fragments with the size of 200–400 bp were selected with beads and hybridized with capture probes baits. After polymerase chain reaction amplification, libraries were sequenced on the NextSeq N500 platform (Illumina, San Diego, USA). Data were further analyzed to identify gene alterations (e.g. *EGFR* mutation, *KRAS* mutation, *ALK* rearrangement, *ROS1* rearrangement, *TP53* mutation).

### Statistical analyses

Data were analyzed by SPSS software (version 22.0, IBM Corp, Armonk, NY) and R (version 3.6.0). The correlations between pathological factors and PD-L1 expression were examined using the Kruskal–Wallis test. Weighted kappa statistic with quadratic weights was used to determine the concordance between two groups. Kappa statistic was further categorized as followed: ≤ 0 = none, 0.01–0.20 = poor, 0.21–0.40 = slight, 0.41–0.60 = fair, 0.61–0.80 = good, 0.81–0.92 = very good, 0.93–1.00 = excellent [7, 8].

## Results

### Patient characteristics and PD-L1 expression

A total of 4550 patients were identified according to the inclusion criteria. Among them, 72 patients received two PD-L1 expression tests, including 47 patients with two surgically-resected lesions (15 patients with second primary lung cancer and 32 patients with intrapulmonary metastasis), 12 patients with paired biopsy and surgical resection specimens, and 13 patients with two biopsies specimens for one lesion. For the rest 4478 patients, most (4320/4478, 96.5%) of them had surgically-resected samples tested, while only 3.5% (158/4479) had biopsy samples tested.

Of the 4550 patients, there were equal proportions of males (51.6%) and females (48.4%). 1815 patients (39.9%) were present or former smokers, while the rest (60.1%) were not. There were 3055 patients (67.1%) with stage 0/I lung cancer, 524 patients (11.5%) with stage II, 773 patients (17.0%) with stage III, and 56 patients (1.2%) with stage IV (Table 1).

**Table 1:** Patients characteristics stratified by PD-L1 expression levels

Variables	All cases (N = 4550)	PD-L1 TPS			*P* values
		PD-L1 < 1% (N = 3017)	1 ≤ PD-L1 ≤ 49% (N = 1013)	PD-L1 ≥ 50% (N = 520)	
Age (years)					P < 0.001
Median (IQR)	62 (55, 68)	62 (54, 67)	62 (55, 68)	64 (58, 69)	
Mean (SD)	61.1 (9.4)	60.7 (9.6)	61.2 (9.3)	62.9 (8.5)	
Range	(17, 85)	(17, 85)	(20, 84)	(34, 83)	
Sex					P < 0.001
Female	2204	1679 (76.2)	404 (18.3)	121 (5.5)	
Male	2346 51	1338 (57.0)	609 (26.0)	399 (17.0)	
Smoking history					P < 0.001
Yes	1815	975 (53.7)	499 (27.5)	341 (18.8)	
No	2735	2042 (74.7)	514 (18.8)	179 (6.5)	
Histology					P < 0.001
AIS/MIA	156	151 (96.8)	5 (3.2)	0 (0)	
IAC	3660	2619 (71.6)	744 (20.3)	297 (8.1)	
SQCC	642	213 (33.2)	229 (35.7)	200 (31.2)	
LCC	49	27 (55.1)	13 (26.5)	9 (18.4)	
ASC	25	1 (4.0)	15 (60.0)	9 (36.0)	
Others	18	6 (33.3)	7 (38.9)	5 (27.8)	
T stage					P < 0.001
T0/1	3022	2262 (74.9)	555 (18.4)	205 (6.8)	
T2	936	443 (47.3)	303 (32.4)	190 (20.3)	
T3	303	155 (51.2)	79 (26.1)	69 (22.8)	
T4	224	126 (56.3)	62 (27.7)	36 (16.1)	
Tx	65	31 (47.7)	14 (21.5)	20 (30.8)	
N stage					P < 0.001
N0	3502	2558 (73.0)	657 (18.8)	287 (8.2)	
N1	323	140 (43.3)	113 (35.0)	70 (21.7)	
N2	679	298 (43.9)	230 (33.9)	151 (22.2)	
N3	40	19 (47.5)	10 (25.0)	11 (27.5)	
Nx	6	2 (33.3)	3 (50.0)	1 (16.7)	
M stage					P < 0.001
M0	4353	2915 (67.0)	966 (22.2)	472 (10.8)	
M1	56	39 (69.6)	9 (16.1)	8 (14.3)	
Mx	141	63 (44.7)	38 (27.0)	40 (28.4)	
TNM stage					P < 0.001
0/IA	2633	2082 (79.1)	423 (16.1)	128 (4.9)	
IB	422	229 (54.3)	121 (28.7)	72 (17.1)	
II	524	247 (47.1)	163 (31.1)	114 (21.8)	
III	773	356 (46.1)	259 (33.5)	158 (20.4)	
IV	56	39 (69.6)	9 (16.1)	8 (14.3)	
x	142	64 (45.1)	38 (26.8)	40 (28.2)	
STAS					P < 0.001
Absence	1927	1555 (80.7)	295 (15.3)	77 (4.0)	
Presence	981	555 (56.6)	301 (30.7)	125 (12.7)	
Unknown	1642	907 (55.2)	417 (25.4)	318 (19.4)	

TPS: tumor proportion score; IQR: interquartile range; SD: standard deviation; AIS: adenocarcinoma in situ; MIA: minimally invasive adenocarcinoma; IAC: invasive adenocarcinoma; SQCC: squamous cell carcinoma; LCC: large cell carcinoma; ASC: adenosquamous carcinoma; STAS: spread through air spaces

According to the percentage of PD-L1 positive tumor cells in all tumor cells, patients were categorized into three groups. 3017 patients (66.3%) had no PD-L1 expression (TPS < 1%), 1013 patients (22.3%) had low PD-L1 expression (TPS 1–49%), and 520 patients (11.4%) had high PD-L1 expression (TPS ≥ 50%) (Table 1). There were significant differences in age (*P* < 0.001), sex (*P* < 0.001), smoking history (*P* < 0.001), histology (*P* < 0.001), TNM stage (*P* < 0.001), and STAS status (*P* < 0.001) among groups with distinct PD-L1 expression. Smokers had greater proportion of high PD-L1 expression (TPS ≥ 50%) than non-smokers (18.8% versus 6.5%) (Table 1).

As for tumor histology, invasive lung adenocarcinoma (LUAD) accounted for 80.4% of cases, whereas squamous cell carcinoma for 14.1%. Squamous cell carcinoma had significantly higher frequency of PD-L1 expression than adenocarcinoma generally (66.9% versus 28.4% of patients with TPS > 1%), while large cell carcinoma had moderate proportion (44.9%) of PD-L1 expression (Table 1). Overall, PD-L1 exhibited higher expression, as patients developed more advanced T stage, N stage, and M stage. High expression of PD-L1 (TPS ≥ 50%) occupied 4.9% of stage 0/IA, 17.1% of stage IB, 21.8% for stage II, and 20.0% for stage III/IV. For cases in which STAS was reported (N = 2908), STAS presence had higher frequency of positive PD-L1 expression (43.4%), compared with STAS absence (19.3%) (Table 1). In order to identify independent predictive factors for high expression of PD-L1 (TPS ≥ 50%), logistic regression analyses were further conducted. The results revealed that sex (*P* = 0.003), smoking history (*P* = 0.039), histology (*P* < 0.001), and TNM stage (*P* < 0.001) could predict high expression of PD-L1 independently (Table 2).

**Table 2:** Univariate and multivariable logistic regression analyses of factors predicting PD-L1 TPS ≥ 50%

Variables	Univariate		Multivariate	
	HR (95% CI)	*P*	HR (95% CI)	*P*
Age	1.025 (1.015, 1.036)	** < 0.001**	1.008 (0.997, 1.020)	0.160
Sex (male VS. female)	3.528 (2.852, 4.364)	** < 0.001**	1.623 (1.181, 2.232)	**0.003**
Smoking history	3.303 (2.727, 4.002)	** < 0.001**	1.362 (1.016, 1.826)	**0.039**
Histology		** < 0.001**		** < 0.001**
Adenocarcinoma	Reference		Reference	
SQCC	5.361 (4.369, 6.580)	** < 0.001**	2.760 (2.174, 3.506)	** < 0.001**
LCC	2.666 (1.281, 5.547)	**0.009**	1.375 (0.645, 2.930)	0.410
ASC	6.665 (2.920, 15.211)	** < 0.001**	4.476 (1.909, 10.498)	**0.001**
Others	4.557 (1.614, 12.870)	**0.004**	3.107 (1.061, 9.100)	**0.039**
TNM stage		** < 0.001**		** < 0.001**
0/IA	Reference		Reference	
IB	4.026 (2.954, 5.487)	** < 0.001**	2.914 (2.110, 4.026)	** < 0.001**
II	5.442 (4.141, 7.151)	** < 0.001**	2.971 (2.212, 3.991)	** < 0.001**
III	5.028 (3.919, 6.451)	** < 0.001**	3.660 (2.822, 4.746)	** < 0.001**
IV	3.262 (1.511, 7.039)	**0.003**	3.706 (1.691, 8.123)	**0.001**
x	7.675 (5.111, 11.524)	** < 0.001**	6.542 (4.300, 9.954)	** < 0.001**

The* P* values in bold indicate statistical significance (*P* < 0.05)

HR: hazard ratio; CI: Confidence interval; SQCC: squamous cell carcinoma; LCC: large cell carcinoma; ASC: adenosquamous carcinoma

### Adenocarcinoma subtypes and PD-L1 expression

**Fig. 1:**
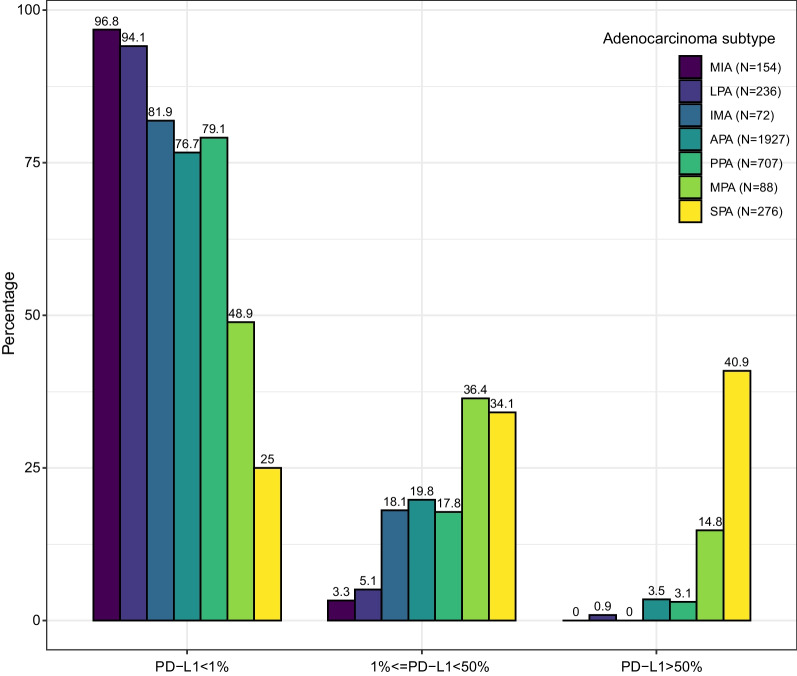
Percentages of PD-L1 expression in patients with distinct adenocarcinoma subtypes. MIA: minimally invasive adenocarcinoma, LPA: lepidic pattern-predominant adenocarcinoma, APA: acinar pattern-predominant adenocarcinoma, PPA: papillary pattern-predominant adenocarcinoma, IMA: invasive mucinous adenocarcinoma, MPA: micropapillary pattern-predominant adenocarcinoma, SPA: solid pattern-predominant adenocarcinoma

According to the adenocarcinoma subtypes released by the International Association for the Study of Lung Cancer, American Thoracic Society, and European Respiratory Society [9], LUAD could be further divided into adenocarcinoma in situ, minimally invasive adenocarcinoma (MIA), lepidic pattern-predominant adenocarcinoma (LPA), acinar pattern-predominant adenocarcinoma (APA), papillary pattern-predominant adenocarcinoma (PPA), invasive mucinous adenocarcinoma (IMA), micropapillary pattern-predominant adenocarcinoma (MPA), and solid pattern-predominant adenocarcinoma (SPA). MIA displayed lowest positive rate (3.3%) of PD-L1, and low-grade LUAD (LPA) also had low expression of PD-L1 (Fig. 1). Intermediate-grade LUAD (IMA, APA, and PPA) was associated with moderate expression of PD-L1, and high expression of PD-L1 was more common in high-grade LUAD (MPA and SPA) (Fig. 1). Taking together, PD-L1 expression was elevated along with the progression of LUAD.

### Concordance of PD-L1 expression in patients with multiple lung cancers

**Fig. 2:**
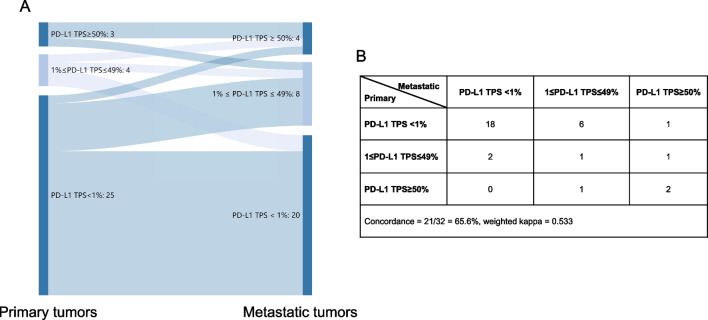
Sankey diagram (**A**) and details (**B**) of PD-L1 expression using 22C3 assays in primary and metastatic tumors. TPS: tumor proportion score

Multiple lung cancers could be multifocal primary lesions or intrapulmonary metastases. In this study, the distinguishment of multiple lesions was based on proposals from IASLC Lung Cancer Staging Project [10]. In 32 cases with paired primary and metastatic tumors, PD-L1 expression revealed fair agreement (overall concordance = 65.6%, weighted kappa = 0.533) (Fig. 2). Most dis-concordance (6/11, 54.5%) occurred in patients with no PD-L1 expression (TPS < 1%) in primary tumors and low PD-L1 expression (TPS 1–49%) in metastatic tumors. In 15 cases with two primary lesions, poor agreement was observed (overall concordance = 66.7%, weighted kappa = 0.045) (Additional file 1: Fig. S1). There were three patients with PD-L1 TPS ≥ 50% in tumor 1 and < 1% in tumor 2, which decreased the weighted kappa at quadratic level.

### Concordance of PD-L1 expression in patients with biopsy/surgical resection or two biopsies

**Fig. 3:**
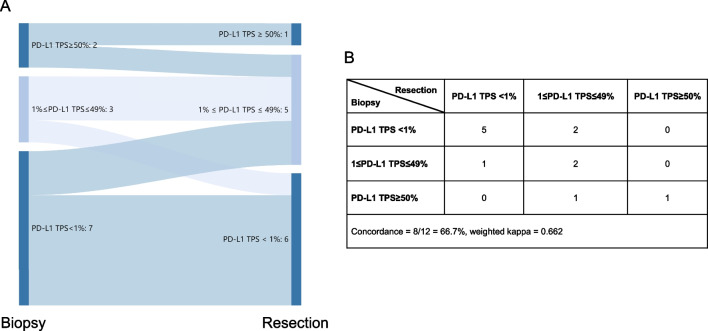
Sankey diagram (**A**) and details (**B**) of PD-L1 expression using 22C3 assays in biopsy and surgically-resected tumors. TPS: tumor proportion score

**Fig. 4:**
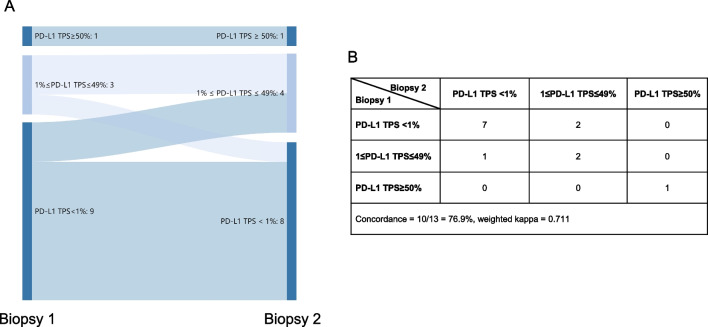
Sankey diagram (**A**) and details (**B**) of PD-L1 expression using 22C3 assays in lung cancer patients receiving two biopsies. TPS: tumor proportion score

Biopsy was considered to be an essential way for diagnosis of lung cancer. 12 patients received biopsy and then surgical resection, and PD-L1 expression in biopsy samples showed good agreement with that in their paired resection samples (overall concordance = 66.7%, weighted kappa = 0.662) (Fig. 3). Of four patients with inconsistent results of PD-L1 expression, three were smoking males. In 13 cases receiving two biopsies, good agreement was revealed (overall concordance = 76.9%, weighted kappa = 0.711) (Fig. 4).

### PD-L1 expression and molecular events

**Fig. 5:**
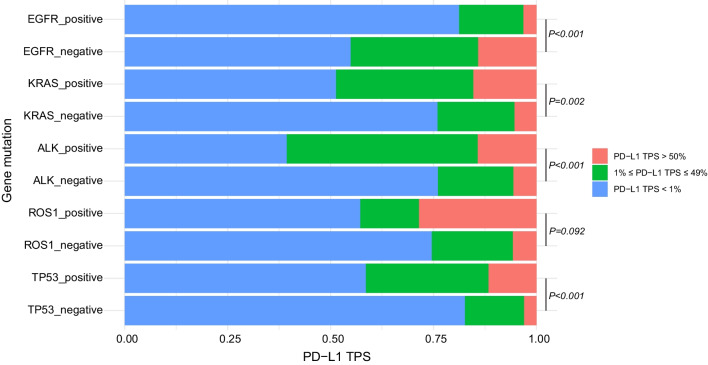
The distribution of PD-L1 expression using 22C3 assays stratified by mutational status of *EGFR*, *KRAS*, *ALK*, *ROS1*, and *TP53*

Since immune and targeted therapies were two significant parts of lung cancer treatments, the association between PD-L1 expression and molecular events was further investigated. Among 596 patients with LUAD receiving 68-gene panel sequencing, 441 (74.0%) harbored *EGFR* mutation, 39 (6.5%) harbored *KRAS* mutation, 28 (4.7%) had *ALK* rearrangement, 7 (1.2%) had *ROS1* rearrangement, and 205 (34.4%) had *TP53* mutation. Tumors with *EGFR* mutation had more frequency in PD-L1 negative expression (TPS < 1%) (81.2% versus 54.8%) and less frequency in PD-L1 high expression (TPS ≥ 50%) (3.2% versus 14.2%) compared with *EGFR* wild-type tumors (*P* < 0.001) (Fig. 5). Nevertheless, *KRAS*-mutant tumors exhibited greater prevalence of PD-L1 high expression (TPS ≥ 50%) (15.4% versus 5.4%) and less prevalence of PD-L1 negative expression (TPS < 1%) (51.3% versus 75.9%) compared with *KRAS* wild-type tumors (*P* = 0.002) (Fig. 5). The results also showed higher proportion of PD-L1 positive expression were associated with *ALK* rearrangement and *TP53* mutation (*P* < 0.001 for *ALK* rearrangement, *P* < 0.001 for *TP53* mutation) (Fig. 5).

## Discussion

ICIs, as monotherapy or in combination with traditional chemotherapy, have become a mainstay of first-line treatments for patients with advanced or metastatic NSCLC [1, 11–17]. The US Food and Drug Administration has approved ICIs for the first-line treatment of NSCLC with specific PD-L1 expression limits (pembrolizumab: TPS ≥ 1%, atezolizumab: TPS ≥ 50%, cemiplimab-rwlc: TPS ≥ 50%), based on previous results of phase 3 randomized controlled trials [12, 15, 18]. Therefore, PD-L1 testing is critical for selection and personalized treatments to lung cancer patients receiving immune therapy. In this study, we reported real-world prevalence of PD-L1 expression using 22C3 assays and its association with common molecular events in mostly surgical-resected specimens from a large-scale East-Asian cohort. Further analyses revealed good agreement in biopsy and resection samples/ two biopsy samples, fair agreement in primary and metastatic lesions, and poor agreement in multi-focal primary tumors. Our study provides evidence for the optimization of PD-L1 testing for lung cancer.

In this study, there were 66.3% of cases with no PD-L1 expression (TPS < 1%), 22.3% with low PD-L1 expression (TPS 1–49%), and 11.4% with high PD-L1 expression (TPS ≥ 50%) generally, which was distinct from Caucasians [19, 20]. The prevalence of high PD-L1 expression is 30–35% in western cases with NSCLC [20–23], whereas it is less than 20% in East-Asian patients [24, 25], which is consistent with our study. The difference could be explained by the high prevalence of *EGFR* mutation and smoking status in East-Asian patients with lung cancer. Previous studies have demonstrated that negative expression of PD-L1 was associated with *EGFR* mutation and no history of smoking [26, 27]. In East-Asian cases, there were higher proportions of patients with *EGFR* mutation and non-smokers compared with those from western countries [28, 29]. Nevertheless, current evidences suggest ICIs might have better efficacy in Asians compared with white patients with NSCLC [30]. KEYNOTE-024 [31] showed East Asians were more sensitive to pembrolizumab than non-East Asians (East Asians, hazard ratio [HR]: 0.35, 0.14–0.91; non-East Asians, HR: 0.52, 0.38–0.72), in spite of all the enrolled patients with high PD-L1 expression (TPS ≥ 50%). In a study investigating atezolizumab plus chemotherapy for non-squamous NSCLC regardless of PD-L1 expression (IMpower132) [32], superior OS was also observed in Asian patients (Asian, hazard ratio [HR]: 0.42, 0.28–0.63; white, HR: 0.67, 0.54–0.84). Future studies are urged to reveal molecular mechanisms behind the differences of ICIs efficacy in distinct races.

Synchronous lung nodules, which could be multifocal primary lesions or intrapulmonary metastasis, have been identified in 3.7–8% of patients [33]. In this study, we reported fair agreement in primary and metastatic lesions (weighted kappa = 0.533) and poor agreement in multi-focal primary tumors (weighted kappa = 0.045). Hwang et al. [20] also reported similar results about heterogeneity of PD-L1 expression in primary and metastatic tumors (weighted kappa = 0.48). As a result, it might be unnecessary for recurrent patients with NSCLC to receive another PD-L1 expression test, if they have received PD-L1 testing for primary tumors.

In addition, good agreement was verified in biopsy and resection samples (weighted kappa = 0.662), which is in line with previous studies investigating the heterogeneity of PD-L1 expression [20, 34]. It indicates that PD-L1 testing for biopsy can represent the whole picture, and the PD-L1 expression from biopsy specimens may be used to guide ICI treatment for NSCLC. Moreover, there was also good concordance between two biopsies. In this study, biopsy sites could be primary tumors or metastatic lymph nodes. Therefore, it is feasible in clinical practice to perform biopsy on any accessible sites.

The association between PD-L1 expression and common gene alterations was also revealed in our study. Similar to previous study [17, 21, 22, 35], patients with wild-type *EGFR* were associated higher expression level of PD-L1 compared with those with *EGFR* mutation, which gives the rationality to immunotherapy for NSCLC patients with wild-type *EGFR*. On the contrary, patients harboring *KRAS* mutation or *ALK* rearrangement might also have significantly higher frequency of positive PD-L1 expression. Recently, adagrasib and sotorasib showed excellent efficacy for NSCLC with *KRAS* G12C mutation [36, 37], and combination of ICIs and *KRAS* mutation might provide better survival than monotherapy.

We acknowledge that there were some limitations and biases this study. First, only 72 enrolled patients received PD-L1 expression tests for two times, because it was determined by experienced pulmonary pathologists whether to perform tests of PD-L1 expression according to different clinical scenarios. Second, given the nature of retrospective study, selection and time-trend bias were inevitable. Our cohort was based on the Chinese population and only included the patients who were hospitalized in the department of thoracic surgery of our institution. Therefore, more than 60% of cases had stage 0/I NSCLC, and 96.5% of PD-L1 expression came from surgically-resected samples, which were considered to be optimal specimen types for the tests of PD-L1 expression.

In summary, our study revealed the unique distribution pattern of PD-L1 expression using the 22C3 assay from 4550 East-Asian patients with NSCLC, which was distinct from Caucasians. We also reported the association between PD-L1 expression and common molecular events and the concordance of PD-L1 expression between synchronous lung nodules (multifocal primary tumors or metastatic tumors), biopsy/resection specimens, and two biopsy specimens. The results provide insight into the optimization of clinical tests of PD-L1 expression using the 22C3 assay.

## Supplementary Information

**Additional file 1: Figure S1.** Sankey diagram (A) and details (B) of PD-L1 expression using 22C3 assays in two multi-focal primary tumors. TPS: tumor proportion score.

## Data Availability

Data sharing is not applicable to this article as no datasets were generated or analysed during the current study.

## Abbreviations

FFPE: Formalin-fixed paraffin-embedded
FUSCC: Fudan University Shanghai Cancer Center
ICIs: Immune-checkpoint inhibitors
NGS: Next-generation sequencing
OS: Overall survival
NSCLC: Non-small cell lung cancer
PD-L1: Programmed death ligand-1
STAS: Tumor spread thorough air space
TPS: Tumor proportion score

## References

[1] Reck M, Rodriguez-Abreu D, Robinson AG, Hui R, Csoszi T, Fulop A, Gottfried M, Peled N, Tafreshi A, Cuffe S (2021). Five-year outcomes with pembrolizumab versus chemotherapy for metastatic non-small-cell lung cancer with PD-L1 tumor proportion score >/= 50. J Clin Oncol.

[2] Lantuejoul S, Sound-Tsao M, Cooper WA, Girard N, Hirsch FR, Roden AC, Lopez-Rios F, Jain D, Chou TY, Motoi N (2020). PD-L1 testing for lung cancer in 2019: perspective from the IASLC Pathology Committee. J Thorac Oncol.

[3] Duchemann B, Remon J, Naigeon M, Cassard L, Jouniaux JM, Boselli L, Grivel J, Auclin E, Desnoyer A, Besse B, Chaput N (2021). Current and future biomarkers for outcomes with immunotherapy in non-small cell lung cancer. Transl Lung Cancer Res.

[4] Memmott RM, Wolfe AR, Carbone DP, Williams TM (2021). Predictors of response, progression-free survival, and overall survival in patients with lung cancer treated with immune checkpoint inhibitors. J Thorac Oncol.

[5] Uruga H, Mino-Kenudson M (2021). Predictive biomarkers for response to immune checkpoint inhibitors in lung cancer: PD-L1 and beyond. Virchows Arch.

[6] Detterbeck FC, Marom EM, Arenberg DA, Franklin WA, Nicholson AG, Travis WD, Girard N, Mazzone PJ, Donington JS, Tanoue LT (2016). The IASLC Lung Cancer Staging Project: background data and proposals for the application of TNM staging rules to lung cancer presenting as multiple nodules with ground glass or lepidic features or a pneumonic type of involvement in the forthcoming eighth edition of the TNM classification. J Thorac Oncol.

[7] Carpenter CR (2005). Kappa statistic. CMAJ.

[8] Byrt T (1996). How good is that agreement?. Epidemiology.

[9] Travis WD, Brambilla E, Noguchi M, Nicholson AG, Geisinger KR, Yatabe Y, Beer DG, Powell CA, Riely GJ, Van Schil PE (2011). International association for the study of lung cancer/american thoracic society/european respiratory society international multidisciplinary classification of lung adenocarcinoma. J Thorac Oncol.

[10] Detterbeck FC, Nicholson AG, Franklin WA, Marom EM, Travis WD, Girard N, Arenberg DA, Bolejack V, Donington JS, Mazzone PJ (2016). The IASLC Lung Cancer Staging Project: summary of proposals for revisions of the classification of lung cancers with multiple pulmonary sites of involvement in the forthcoming eighth edition of the TNM classification. J Thorac Oncol.

[11] West H, McCleod M, Hussein M, Morabito A, Rittmeyer A, Conter HJ, Kopp HG, Daniel D, McCune S, Mekhail T (2019). Atezolizumab in combination with carboplatin plus nab-paclitaxel chemotherapy compared with chemotherapy alone as first-line treatment for metastatic non-squamous non-small-cell lung cancer (IMpower130): a multicentre, randomised, open-label, phase 3 trial. Lancet Oncol.

[12] Socinski MA, Jotte RM, Cappuzzo F, Orlandi F, Stroyakovskiy D, Nogami N, Rodriguez-Abreu D, Moro-Sibilot D, Thomas CA, Barlesi F (2018). Atezolizumab for first-line treatment of metastatic nonsquamous NSCLC. N Engl J Med.

[13] Paz-Ares L, Luft A, Vicente D, Tafreshi A, Gumus M, Mazieres J, Hermes B, Cay Senler F, Csoszi T, Fulop A (2018). Pembrolizumab plus chemotherapy for squamous non-small-cell lung cancer. N Engl J Med.

[14] Gandhi L, Rodriguez-Abreu D, Gadgeel S, Esteban E, Felip E, De Angelis F, Domine M, Clingan P, Hochmair MJ, Powell SF (2018). Pembrolizumab plus chemotherapy in metastatic non-small-cell lung cancer. N Engl J Med.

[15] Mok TSK, Wu Y-L, Kudaba I, Kowalski DM, Cho BC, Turna HZ, Castro G, Srimuninnimit V, Laktionov KK, Bondarenko I (2019). Pembrolizumab versus chemotherapy for previously untreated, PD-L1-expressing, locally advanced or metastatic non-small-cell lung cancer (KEYNOTE-042): a randomised, open-label, controlled, phase 3 trial. Lancet.

[16] Horn L, Mansfield AS, Szczesna A, Havel L, Krzakowski M, Hochmair MJ, Huemer F, Losonczy G, Johnson ML, Nishio M (2018). First-line atezolizumab plus chemotherapy in extensive-stage small-cell lung cancer. N Engl J Med.

[17] Antonia SJ, Borghaei H, Ramalingam SS, Horn L, De Castro CJ, Pluzanski A, Burgio MA, Garassino M, Chow LQM, Gettinger S (2019). Four-year survival with nivolumab in patients with previously treated advanced non-small-cell lung cancer: a pooled analysis. Lancet Oncol.

[18] Sezer A, Kilickap S, Gumus M, Bondarenko I, Ozguroglu M, Gogishvili M, Turk HM, Cicin I, Bentsion D, Gladkov O (2021). Cemiplimab monotherapy for first-line treatment of advanced non-small-cell lung cancer with PD-L1 of at least 50%: a multicentre, open-label, global, phase 3, randomised, controlled trial. Lancet.

[19] Saez de Gordoa K, Lopez I, Marginet M, Coloma B, Frigola G, Vega N, Martinez D, Teixido C (2021). PD-L1 expression in non-small cell lung cancer: data from a referral center in Spain. Diagnostics.

[20] Hwang DM, Albaqer T, Santiago RC, Weiss J, Tanguay J, Cabanero M, Leung Y, Pal P, Khan Z, Lau SCM (2021). Prevalence and heterogeneity of PD-L1 expression by 22C3 assay in routine population-based and reflexive clinical testing in lung cancer. J Thorac Oncol.

[21] Dietel M, Savelov N, Salanova R, Micke P, Bigras G, Hida T, Antunez J, Guldhammer Skov B, Hutarew G, Sua LF (2019). Real-world prevalence of programmed death ligand 1 expression in locally advanced or metastatic non-small-cell lung cancer: the global, multicenter EXPRESS study. Lung Cancer.

[22] Evans M, O'Sullivan B, Hughes F, Mullis T, Smith M, Trim N, Taniere P (2020). The clinicopathological and molecular associations of PD-L1 expression in non-small cell lung cancer: analysis of a series of 10,005 cases tested with the 22C3 assay. Pathol Oncol Res.

[23] Holmes M, Mahar A, Lum T, Kao S, Cooper WA (2021). Real-world programmed death-ligand 1 prevalence rates in non-small cell lung cancer: correlation with clinicopathological features and tumour mutation status. J Clin Pathol.

[24] Saito T, Tsuta K, Ishida M, Ryota H, Takeyasu Y, Fukumoto KJ, Matsui H, Taniguchi Y, Yanagimoto H, Kurata T, Murakawa T (2018). Comparative study of programmed cell death ligand-1 immunohistochemistry assays using 22C3 and 28–8 antibodies for non-small cell lung cancer: analysis of 420 surgical specimens from Japanese patients. Lung Cancer.

[25] Xu Z, Li H, Dong Y, Cheng P, Luo F, Fu S, Gao M, Kong L, Che N (2020). Incidence and PD-L1 expression of MET 14 skipping in Chinese population: a non-selective NSCLC cohort study using RNA-based sequencing. Onco Targets Ther.

[26] Takada K, Toyokawa G, Tagawa T, Kohashi K, Shimokawa M, Akamine T, Takamori S, Hirai F, Shoji F, Okamoto T (2018). PD-L1 expression according to the EGFR status in primary lung adenocarcinoma. Lung Cancer.

[27] Norum J, Nieder C (2018). Tobacco smoking and cessation and PD-L1 inhibitors in non-small cell lung cancer (NSCLC): a review of the literature. ESMO Open.

[28] Fukuoka M, Wu YL, Thongprasert S, Sunpaweravong P, Leong SS, Sriuranpong V, Chao TY, Nakagawa K, Chu DT, Saijo N (2011). Biomarker analyses and final overall survival results from a phase III, randomized, open-label, first-line study of gefitinib versus carboplatin/paclitaxel in clinically selected patients with advanced non-small-cell lung cancer in Asia (IPASS). J Clin Oncol.

[29] Scagliotti GV, Longo M, Novello S (2009). Nonsmall cell lung cancer in never smokers. Curr Opin Oncol.

[30] Qian J, Nie W, Lu J, Zhang L, Zhang Y, Zhang B, Wang S, Hu M, Xu J, Lou Y (2020). Racial differences in characteristics and prognoses between Asian and white patients with nonsmall cell lung cancer receiving atezolizumab: an ancillary analysis of the POPLAR and OAK studies. Int J Cancer.

[31] Reck M, Rodriguez-Abreu D, Robinson AG, Hui R, Csoszi T, Fulop A, Gottfried M, Peled N, Tafreshi A, Cuffe S (2016). Pembrolizumab versus chemotherapy for PD-L1-positive non-small-cell lung cancer. N Engl J Med.

[32] Nishio M, Barlesi F, West H, Ball S, Bordoni R, Cobo M, Longeras PD, Goldschmidt J, Novello S, Orlandi F (2021). Atezolizumab plus chemotherapy for first-line treatment of nonsquamous NSCLC: results from the randomized phase 3 IMpower132 trial. J Thorac Oncol.

[33] Gazdar AF, Minna JD (2009). Multifocal lung cancers–clonality vs field cancerization and does it matter?. J Natl Cancer Inst.

[34] Kitazono S, Fujiwara Y, Tsuta K, Utsumi H, Kanda S, Horinouchi H, Nokihara H, Yamamoto N, Sasada S, Watanabe S (2015). Reliability of small biopsy samples compared with resected specimens for the determination of programmed death-ligand 1 expression in non–small-cell lung cancer. Clin Lung Cancer.

[35] Boothman AM, Scott M, Ratcliffe M, Whiteley J, Dennis PA, Wadsworth C, Sharpe A, Rizvi NA, Garassino MC, Walker J (2019). Impact of patient characteristics, prior therapy, and sample type on tumor cell programmed cell death ligand 1 expression in patients with advanced NSCLC screened for the ATLANTIC Study. J Thorac Oncol.

[36] Awad MM, Liu S, Rybkin II, Arbour KC, Dilly J, Zhu VW, Johnson ML, Heist RS, Patil T, Riely GJ (2021). Acquired resistance to KRAS(G12C) inhibition in cancer. N Engl J Med.

[37] Skoulidis F, Li BT, Dy GK, Price TJ, Falchook GS, Wolf J, Italiano A, Schuler M, Borghaei H, Barlesi F (2021). Sotorasib for lung cancers with KRAS p.G12C mutation. N Engl J Med.

