# Supplementary material for: Distribution and concordance of PD-L1 expression by routine 22C3 assays in East-Asian patients with non-small cell lung cancer
Source: Respir Res. 2022 Nov 5;23:302. doi: 10.1186/s12931-022-02201-8 (PMC9636784; doi:10.1186/s12931-022-02201-8)
Supplement: Supplementary file 1 — Additional file 1: Figure S1. Sankey diagram (A) and details (B) of PD-L1 expression using 22C3 assays in two multi-focal primary tumors. TPS: tumor proportion score. [file 12931_2022_2201_MOESM1_ESM.pdf]

A

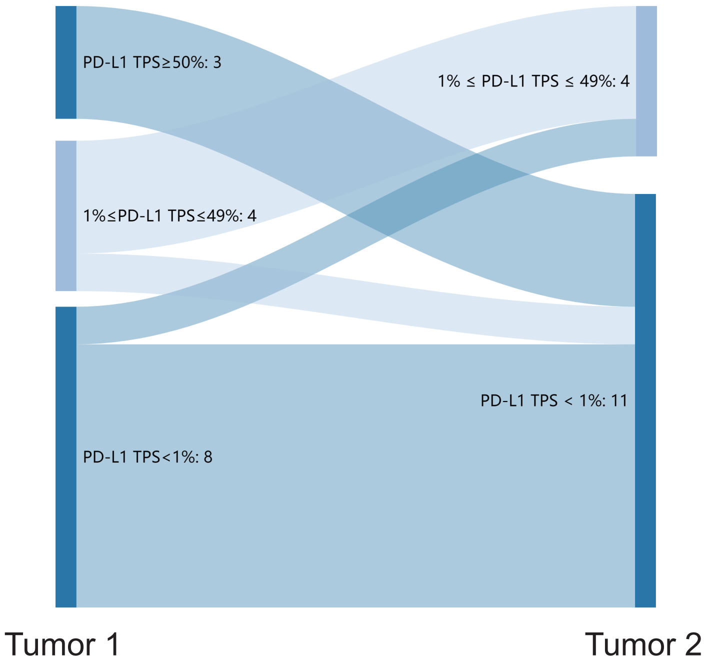

B

Tumor 2 / Tumor 1	PD-L1 TPS <1%	1≤PD-L1 TPS≤49%	PD-L1 TPS≥50%
**PD-L1 TPS <1%**	7	1	0
**1≤PD-L1 TPS≤49%**	1	3	0
**PD-L1 TPS≥50%**	3	0	0
Concordance = 10/15 = 66.7%, weighted kappa = 0.045			
